# An assessment of voting knowledge and related decisions amongst hospitalised mental healthcare users in South Africa

**DOI:** 10.4102/sajpsychiatry.v27i0.1529

**Published:** 2021-01-29

**Authors:** Felicity Marcus, Yvette Nel

**Affiliations:** 1 Department of Psychiatry, Faculty of Health Sciences, University of the Witwatersrand, Johannesburg, South Africa

**Keywords:** CAT-V questionnaire, cognitive assessment tool, doe scoring, schizophrenia, voting

## Abstract

**Background:**

The South African Constitution protects the right to vote for every citizen. The *Electoral Act* (No. 73 of 1998) limits registration on the voter’s roll on the basis of being declared of ‘unsound mind’ or ‘mentally disordered’ by the high court or detention under the *Mental Health Care Act* (No. 17 of 2002). There is limited information regarding voting knowledge and subsequent voting-related decisions amongst South African involuntary mental healthcare users (MHCUs).

**Aim:**

To compare voting knowledge and related decisions between hospitalised MHCUs and non-psychiatric hospitalised patients (controls).

**Setting:**

Participants were recruited from Sterkfontein Psychiatric Hospital (MHCUs) and Chris Hani Baragawanth Academic Hospital orthopaedic wards (controls) in Gauteng, South Africa.

**Method:**

A cross-sectional survey was conducted using a modified Cognitive Assessment Tool for Voting (MCAT-V) questionnaire. Scores on the MCAT-V were compared between the MHCU and control groups, along with socio-demographic variables and clinical variables.

**Results:**

There was a significant association between group (MHCU vs. control) and HLOE (*p* = 0.016). Although the median overall score for the controls (11; interquartile range [IQR] 10–12) was significantly higher than that for the MHCUs (10; IQR 8–12) (*p* = 0.043), when controlling for education level, there was no significant association between group (MHCU/control) and MCAT-V scores (*p* = 0.011). The MCAT-V scores of the ‘Doe questions’ between the MHCUs and controls were not significantly different (*p* = 0.063). There was a difference in ‘reasoning scores’ between MHCUs and controls (*p* = 0.0082) and this was associated with level of educational attainment (*p* = 0.013).

**Conclusion:**

The limitations regarding voter registration legislated in the South African *Electoral Act*, are not supported by the findings of this study. The MCAT-V demonstrates a possible educational bias and therefore is not recommended as a screening tool for assessing voting competency.

## Introduction

‘Universal adult suffrage, a national common voter roll, regular elections and a multi-party system of democratic government’ are a few of the fundamental principles mentioned in the 1996 Constitution of South Africa.^1^ The ability to vote reflects on one’s dignity, equality and freedom of speech within society. The current *Electoral Act* (No. 73 of 1998) sets limitations on certain mental healthcare users (MHCUs) to register to vote in the South African General elections.^2^ Despite numerous amendments of the *Electoral Act* of 1998, with the most recent publication being made in 2019, regulations pertaining to the voting rights of South Africa’s MHCUs have not been revised.^3^

The transgression of human rights with regard to psychiatric patients is not a new concept.^4^ Bhugra et al. conducted a comparative review of the legislations of various countries to assess the rights of mental healthcare individuals to vote.^5^ Of the United Nations Member States studied, 167 of the 193 legislations examined made specific provisions regarding voting in those with psychiatric conditions. It was found that only 21 countries had no restrictions on voting for MHCUs, whilst over one-third of the countries reviewed denied all MHCUs the right to vote. A further 21 countries limited voting in detained psychiatric persons of which nine countries required them to be specifically detained under mental healthcare law. It was found that this exclusion was based on the assumption that psychiatric patients have impaired capacity for voting.^5^

There is evidence to suggest that despite being mentally unwell, MHCUs still retain their political sense.^6^ In Canada, Bhopal et al. looked at the political awareness of hospitalised involuntary patients with chronic psychiatric illnesses. Their study found that 60% of the patients interviewed had a good knowledge of their country’s upcoming elections, and most of these patients were aware of current political issues and situations.^6^

The concept of ‘capacity’ is complex and task specific, and thus there are many ethical and legal factors to consider when attempting to address the idea of voting capacity.^7^ When summarising the international legislation regarding voting rights and limitations in MHCUs, there are various viewpoints: (1) there should be no restrictions on their voting rights in MHCUs; (2) that all MHCUs should be barred from the voting process; and (3) there should be rational legal voting limitations on certain MHCUs.^5^

The current South African *Electoral Act* denies ‘detained’ MHCUs and those who have been declared by the high court to be of ‘unsound mind’ the ability to be registered on the national voters roll and subsequently vote.^2^ There is, however, no adequate clarification of these terms and no means of assessing individual voting competency for those who fall under these categories.^2^

There can be no single test to determine ‘voting competency’; however, it is possible to examine individual voting knowledge and decision-making.^8^ In 2001, in the United States of America, the legal case of Doe versus Rowe resulted in the development of the ‘Doe voting capacity standard’. The United States court ruled that one has the inability to vote only if ‘they lack the capacity to understand the nature and effect of voting such that they cannot make an individual choice’. Appelbaum et al. subsequently created a questionnaire, the Competence Assessment Tool for Voting (CAT-V), based on the Doe standard.^8^ The questionnaire asks the subject to imagine that it is election day, and they must participate in selecting a new governor. A series of three questions based on the Doe criteria are then asked, which assess understanding of the nature and effect of voting and the ability to make a choice. The questionnaire asks a further three questions to assess reasoning regarding the choice of governor. Appelbaum et al. used the CAT-V to assess the voting ability of 33 patients with mild to severe Alzheimer’s disease in an outpatient clinic. The severity of dementia was objectively assessed by using the Mini Mental Status Examination (MMSE). Their study found that those patients with severe dementia scored two or less on the Doe questions, those with moderate dementia obtained variable scores between two and six and all those with mild Alzheimer’s disease achieved a score of six. They concluded that those with mild Alzheimer’s disease still maintained the ability to vote, whereas those with severe Alzheimer’s did not.

Those with moderate Alzheimer’s displayed variable scores, and thus screening tools such as the CAT-V were suggested as useful when assessing their voting competency.^8^

Israeli Election Law makes provisions for hospitalised psychiatric patients to participate in the voting process.

Doron et al.^9^ conducted a study in Israel by utilising the CAT-V questionnaire to assess the relative capability of 56 psychiatric inpatients compared with 12 healthy control subjects to vote. Participants were grouped into high and low capacity to vote by using cluster analysis techniques and the CAT-V score as a continuous measure. Those who obtained a score of ˂ 1 on all six CAT-V questions were considered to have low capacity. Participants who obtained scores of 1.6 or more were considered to have high capacity. They found that 59% of the participants (12 healthy control subjects plus 33 psychiatric patients) had a high capacity to vote. It was found that 41% of psychiatric patients fell into the low-capacity group. When comparing CAT-V scores to the scores obtained on the MMSE, it was found that a direct relationship existed, thus leading them to conclude that the better one’s cognitive functioning, the more likely it is that they will have voting competency. Findings also showed that lower CAT-V scores were associated with a higher Brief Psychiatric Rating Scale (BPRS) score, indicating an association between CAT-V scores and illness severity. It was recommended that the CAT-V could be used as a screening tool for those individuals whose capacity to vote was uncertain.^9,10^

Given the current legislation in South Africa, which at present limits voting registration in persons detained under the MHCA, the focus of this study was to assess voting knowledge and related voting decisions in hospitalised MHCUs in South Africa.

## Objectives

We aimed to compare voting knowledge and related decisions between a group of MHCU and controls, by using a modified CAT-V (MCAT-V) screening tool. We sought to determine if there were any significant differences between the two groups and to determine if there were any additional clinical or other factors, which might influence test scores in the South African context.

## Method

### Participants

This study was conducted as a cross-sectional comparative survey. Data were collected between January 2018 and April 2018. Convenience sampling was used. The MHCU group consisted of 60 patients (26 involuntary MHCUs and 34 state patients) from the psychiatric wards at Sterkfontein Psychiatric Hospital (SPH). A state patient is an individual who has committed an offence of a serious nature and has been deemed by a High Court as suffering from a mental disorder and found therefore not fit to stand trial and/or not responsible for the offence and therefore requiring treatment in a psychiatric hospital.^11^ Involuntary and state patients were selected as opposed to assisted patients as their mental healthcare status was deemed to be synonymous with the patient criteria mentioned in the current *Electoral Act*, although it is not entirely clear which MHCUs the *Electoral Act* is referring to.^2^ The control group comprised 30 hospitalised patients from the orthopaedic wards at Chris Hani Baragwanath Academic Hospital (CHBAH). The sample size was calculated by using the Wilcoxon rank-sum test. It was determined that the sample size of the psychiatric patients would be twice that of the control group, as the psychiatric group was the focal point of the study.^12^ The loss of statistical power from the unequal group size was minimal and was accounted for in the sample size calculation.^12^ Sample size calculations were carried out in G*Power.^13^

### Questionnaire

The CAT-V questionnaire needed to be adapted for the South African population. The unique political, cultural and social situation in South Africa was considered to ensure that the questionnaire would be unbiased and coherent. Permission was obtained to use and modify the CAT-V questionnaire to the South African setting. The MCAT-V required the participant to envision it was the day of the national South African elections. They were obligated to vote for a new political party, not a governor scenario as in the original CAT-V questionnaire.^8^ The question assessing the ability to ‘choose’ was also adjusted to South African circumstances. Candidates were asked to choose between political party A or B, with party A offering monthly grant payouts and party B offering house allowances. These first three questions are considered the ‘Doe Questions’, as they assess the understanding of the nature and effect of voting and the ability to make a choice.^8^ Questions testing the ability to substantiate and reason their choice as well as incentive to vote again remained unchanged (see Appendix). Differences between political party A and B in the hypothetical scenarios were kept vague, with an attempt to avoid any similarities with real political parties and agendas.

### Procedure

Participants were required to understand and speak English, be over the age of 18 years and to hold a valid South African identity document. Participants in the MHCU group were required to be admitted under the *Mental Care Act* 17 of 2002 as an involuntary MHCU or state patient. The control group participants had to be hospitalised for a general orthopaedic condition. Vital signs and physical state were screened to exclude delirium. It was also ensured that the control group had no history of recent head injury (within the last 6 months) and no known psychiatric illness. The usage of benzodiazepines within 8 hours of the interview excluded patients from participating in the study in both the groups.

Informed consent was secured from each candidate prior to participation in the study. The MCAT-V questionnaire was administered by the same investigator for all the participants, and the participant’s answers and scores were documented. Scoring was rated from 0 to 2 points, where 0 was allocated for an inadequate answer, 1 point for a partially correct answer and 2 points for a satisfactory answer. Information pertaining to their allocated ward, the mental healthcare act section under which the user was detained and their diagnosis based on DSM 5 criteria was obtained from hospital records. Age, gender, highest level of education (HLOE) and prior participation in voting (voting status) were captured during the interview.

Contact details of the Independent Electoral Committee were made available to those wishing to enquire about the South African voting process. Provisions were made for referral to the psychology departments of the respective hospitals for participants who were unsettled by the interview process and politically associated questions.

### Data analysis

Data analysis was carried out by using SAS version 9.4 for Windows. The 5% significance level was used.

Descriptive analysis of the data was carried out. An overall score for each participant was calculated (range: 0–12).

The gamma coefficient was used when looking at the association between the questionnaire scores for the control and sample groups as well as the strength of this association. A ‘Doe score’ (sum of question 1–3) and a ‘reasoning score’ (sum of questions 4–6) were calculated for each participant. The chi-squared test was used when calculating the association between gender, HLOE, voting status, categorised scores and groups.

Fisher’s exact test was applied when the requirements for the chi-squared test could not be met. The significance of the association between the two groups was then further calculated by Cramer’s V and the phi coefficient. The association between HLOE and voting status was calculated in a similar manner. The relationship between age, total score and group was analysed by using the *t*-test. Where the data did not meet the assumptions of these tests, the Wilcoxon rank-sum test was used. The strength of the associations was measured by the Cohen’s *d* for parametric tests and the *r* value for the non-parametric tests.

### Ethical consideration

This study was approved by the University of the Witwatersrand post-graduate committee and the Human Research Ethics Committee. Permission was also obtained from SPH and CHBAH. The specialist psychiatrist from each ward involved in the study oversaw the consenting process in the MHCU group (Ethical clearance number: M170730).

## Results

**FIGURE 1:**
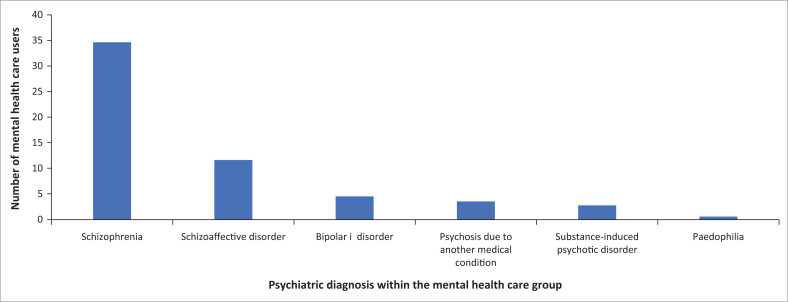
Distribution of the various psychiatric diagnoses based on (Diagnostic and Statistical Manual of Mental Disorders, 5th Edition) DSM 5 criteria within the mental healthcare group (*n* = 60).

There were 90 participants in this study. All participants consented and answered the questionnaire. No patients refused to participate. Of the sample MHCU group, 57% were state patients; the rest (43%) were involuntary MHCUs. The predominant DSM 5 diagnosis in the MHCU group was found to be schizophrenia (58%) (Figure 1). The control group had a diverse range of injuries, with 83% having incurred fractural damage. The participants were all male, and the mean ages of the two groups did not differ significantly (*p* = 0.43) (Table 1). There was a significant association between group and HLOE (*p* = 0.0016) (Table 1); the effect size was moderate (Cramér’s V = 0.38). The control group had a higher proportion of patients with Grade 12 qualification or higher education (63%) compared with the mental healthcare group (25%) (Table 1). There was no significant difference in the percentage of patients who had voted before in the control group (67%) compared with the mental healthcare group (57%) (*p* = 0.49) (Table 1).

**TABLE 1:** Comparison of demographic and clinical data in the control group versus mental healthcare group.

Category	Control group *N* = 30	%	Mental healthcare user group *N* = 60	%	*p*-value for between group test
**Gender**					
Male	30	100	60	100	-
**Age (years)**					0.4300
Mean ± SD	36.3 ± 13.2	-	38.2 ± 8.6	-	
**Level of education**					0.0160†
Special schooling or ≤ Grade 7	2	7	13	22	
Grade 8–11	9	30	32	53
≥ Grade 12	19	63	15	25
**Voted before**					0.4900
Yes	20	67	34	57	
No	10	33	26	43
**MCAT-V overall score**					0.1700
0–5	0	0	6	10	
6–12	30	100	54	90
**Total MCAT-V score**					0.0430†‡
Median ± IQR	11 ± 10–12	-	10 ± 8–12	-	
**MCAT-V ‘Doe scores’**					0.0630 0.6300
0	0	0	1	2	
1–2	0	0	9	15
3–4	8	27	10	17
5–6	22	73	40	67
**MCAT-V ‘reasoning scores’**					0.0082†
0–4	2	7	19	32	
4–6	28	93	41	68

Note: *p*-values calculated by using chi-squared test unless otherwise stated. MCAT-V, Modified Cognitive Assessment Tool for Voting; IQR, interquartile range; SD, standard deviation.

† , Significant (*p* < 0.05).

‡ , Fisher’s exact test.

There was no significant association between HLOE and voting status (*p* = 0.23).

Considering the categorised overall score, all control group participants (100%) scored six or more, compared with only 90% of the MHCU group. The difference, however, was not statistically significant (*p* = 0.17) (Table 1). The median overall score for the control group (11; interquartile range [IQR] 10–12) was significantly higher than that for the MHCU group (10; IQR 8–12) (*p* = 0.043) (Table 1). However, the effect size was small (*r* = 0.22). There was a difference in median scores between involuntary MHCUs (10; IQR 7–11) and state patients (11; IQR 9–12); however, this difference was not significant (*p* = 0.19). No further analysis was conducted on the MHCU subgroups.

When assessing categorised ‘Doe scores’ between the control and MHCU group, the difference was not found to be significant (*p* = 0.063) (Table 1). The proportion of scores five or greater on the Doe questions also did not differ significantly between the two groups (*p* = 0.63) (Table 1). A significant difference was found between the categorised reasoning scores of the two groups (*p* = 0.0082) (Table 1). The proportion of patients with high reasoning scores (i.e. scores of either five or six) was lower in the MHCU group (68%) compared with the control group (93%) (Table 1).

**FIGURE 2:**
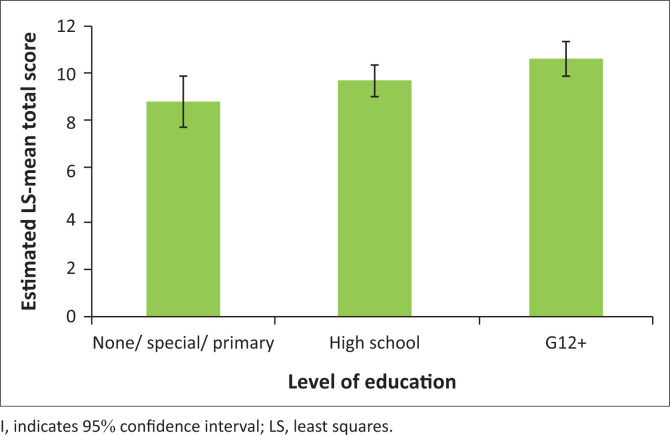
Post-hoc testing of the estimated mean total Modified Cognitive Assessment Tool for Voting scores with associated level of education.

**FIGURE 3:**
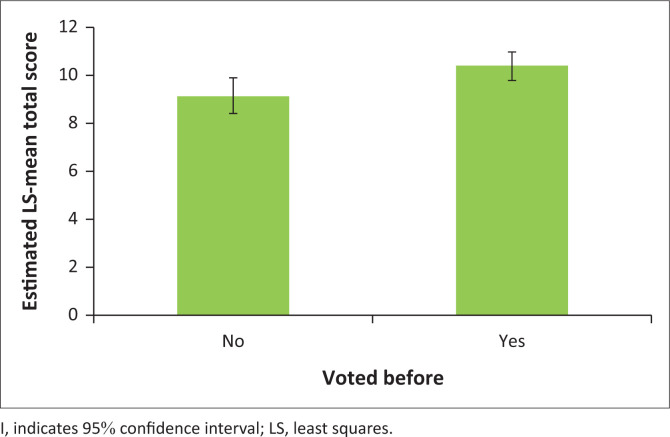
Post-hoc testing of the estimated mean total Cognitive Assessment Tool for Voting scores for those who had voted before compared with those who had not.

On further analysis of the scores and confounding factors, it was found that the HLOE (*p* = 0.020) and voting status (*p* = 0.0051) had a significant effect on the overall MCAT-V score but group did not (*p* = 0.41) (Table 2). Post-hoc tests showed that the estimated mean total score, across groups and voting status, was significantly higher for those with Grade 12 or higher education compared with those with no schooling/special schooling or primary-level education (*p* = 0.017) (Figure 2). Similarly, post-hoc tests revealed that the estimated mean total score, across groups and HLOE, was significantly higher for those who had voted before compared with those who had not (*p* = 0.0051) (Figure 3).

**TABLE 2:** Determination of the association between Modified Cognitive Assessment Tool for Voting overall score and group, highest level of education and voting status.

Source	Degrees of freedom	Type III sum of squares	Mean square	*F*	*p*
Group	1	2.57	2.57	0.70	0.41
HLOE	2	30.32	15.16	4.11	0.020†
Voting status	1	30.58	30.58	8.29	0.0051^‡^

Note: *p*-values calculated by using the General Linear Model.

HLOE, highest level of education.

† , Significant (*p* < 0.05).

‡ , Significant (*p* < 0.01).

## Discussion

The predominant diagnosis amongst the mental healthcare participants in this study was found to be schizophrenia. This was comparable with the study by Raad et al., who reported that 72% of their sample group had a primarily psychotic diagnosis^10^ as well as the study by Doron et al. in which 78% of the inpatient participants met DSM-IV criteria for schizophrenia.^9^ Psychosis alone does not correlate to incapacity.^14^

In the 2016 Annual Meeting of the American Psychiatry Association, it was construed that despite the cognitive impairments experienced by patients suffering from mental illnesses, such as schizophrenia, the ability to make insightful decisions and choices is retained.^14^ Jeste et al. also concluded that a diagnosis of schizophrenia does not equate to incapacity.^15^

No significant difference was found between the Doe scores of the MHCU group and the control group (*p* = 0.063). The percentage of scores of 5 or greater also did not differ significantly between the two groups (*p* = 0.63). Involuntary and state psychiatric patients thus retained their understanding of voting and demonstrated an ability to make a choice despite current hospitalisation for mental illness, relative to a control group. This finding does not support the suggestion that voting knowledge and choice are determined by hospitalisation for psychiatric illnesses. The reasoning and appreciation questions (i.e. questions 4, 5 and 6) showed greater variability between the two groups. Mental healthcare users obtained lower scores than controls. Similar findings were found by Applebaum et al. with regard to CAT-V score patterns in patients with very mild to mild Alzheimer’s disease.^8^ Although participants may hold an understanding and knowledge of the nature and effect of voting and can make a choice, their ability to reason may be compromised.^8^

A significant difference in level of education was found in our study between the control group and the MHCUs with 63% of the control group completing at least 12 years of schooling versus 25% of the MHCU group. A higher number of MHCUs (22%) obtained special schooling or a Grade 7 or less (*p* = 0.0016). This pattern of education is not consistent with previous research in hospitalised MHCUs in South Africa, where it was noted that 63% of acute male admissions in Lentegeur Psychiatric Hospital in the Western Cape Province were educated to a secondary level.^16^ When controlling for differences in level of education, there was no significant association with MCAT-V scores and group (MHCU vs. controls), suggesting that education level influenced total scores on the MCAT-V rather than membership of the group (MHCU vs. control). No previous associations have been reported between CAT-V scores and level of education.^8,9,10^

Previous research on the use of the CAT-V has been conducted in population groups with relatively high levels of education with Applebaum et al. reporting a mean of 14.0 years of education in patients with Alzheimer’s disease, and Doron et al. reporting that 89.9% of MHCUs in their study had a minimum of a high school level of education.^8,9^ In the United States of America, 88% of the population have at least a high school diploma (at least 12 years of education).^17^ In South Africa, it is estimated that only 40% – 50% of learners who start school complete 12 years of education.^18^ It has been found that educational factors impact on test scores in general.^19^ Examinations test the abilities and academic knowledge of the candidate. They also assess skill and task performance with the conditions being decided on by the overseeing examiner. This leads to limitations in assessment and bias.^19^ Competence Assessment Tool for Voting scores may thus be sensitive to level of education, and this association has not been previously detected due to higher average levels of education in previous sample groups. Education is not a requirement for voting capacity in a democracy; therefore, this suggested educational bias limits the usefulness of the MCAT-V as a screening tool in any democracy.

It is also noted that despite current restrictions on voting registration in South Africa, there was no difference in previous participation in the voting process between the two study groups (*p* = 0.49). The timeline of hospitalisation and voting history was not obtained. Previous participation in voting in an election is thought to have a strong influence on one’s ability to vote in future.^20^ Mental healthcare users and control group participants in our study who had voted before were found to have higher mean MCAT-V scores. They were able to adequately explain the voting process and also had experience in the aspect of making informed decisions and choices.

## Study limitations

This study covered a small number of involuntary MHCUs and state patients and was also restricted in that all MHCU participants were admitted at Sterkfontein Psychiatric Hospital (SPH). Results can therefore not be generalised to the South African psychiatric population. This study used convenience sampling, which is also a limitation.

The sample group consisted of both involuntary MHCUs and state patients, and although there was no significant difference in median MCAT-V scores between the two MHCU sub-groups, further differences between these two groups were not explored. The duration of hospital admission between the involuntary users versus state patients may differ, as state patients would have a longer admission duration than involuntary MHCUs, and this could have an impact on mental state, acute illness severity and the subsequent test results.^21^ Duration of hospitalisation was not determined in either MHCU groups. DSM 5 diagnosis used was based on clinical assessment documented in the clinical file, and no rating scales or confirmation of diagnosis was carried out. The illness severity of the psychiatric participants was not assessed, but long-term state patients might be more psychiatrically stable than acutely ill involuntary MHCUs based on the duration of hospitalisation as well as discharge procedures. A long-term follow-up study performed at SPH has shown that after 3 years a large percentage of state patients were clinically stable and able to reside in their communities.^22^ It is also possible that the cognitive profile and premorbid level of functioning might be different between state patients and involuntary MHCUs. It is not possible to confirm from this study that involuntary MHCUs alone would have demonstrated the above patterns of detailed scoring and associations on the MCAT-V.

Objective cognitive testing (such as the MMSE and MOCA) was not carried out as compared with other similar international studies. This added a further limitation to the study as these screening tools may have provided validity for the MCAT-V questionnaire. Cognitive impairment is common in those with severe psychiatric illnesses and may serve as an independent factor when assessing voting capability.^23^ An Italian study, which assessed voting competency in patients with Alzheimer ’s disease, made use of the MMSE, which is a standardised and globally accepted test to measure cognitive ability.^24^ However, MMSE scores were compared with the CAT-V scores and not found to be a firm predictor of voting ability.

It was noted that despite a proportion of MHCUs having a Grade 7 level of education or less, intellectual disability was not mentioned as a DSM 5 diagnosis. Whilst level of education was obtained from the patient directly during the interview, the DSM 5 diagnoses were captured from the patients’ files. This serves as a limitation with regard to discrepancies with diagnosis and possibly indicates poor record-keeping of long-term patients.

Control for level of education by the usage of patients in both groups who obtained a matric certificate or higher may have clarified the impact of MHCU status on MCAT-V scores. However, the finding of a possible educational bias in the MCAT-V is an important finding as it limits the usefulness of the MCAT-V as a screening tool.

The MCAT-V was adjusted for our South African population. However, the reliability and validity of this modification were not confirmed. The adjustment in the first three ‘Doe’ questions was to replace the usage of the word ‘governor’ with that of ‘party’ as it was thought to be a more relatable term to the South African population. Choice of replacement questions and situations were further carried out for the ‘reasoning’ questions 4–6 to assist the South African participants to identify with the questionnaire. The attempts were to retain the basic ‘Doe principles’ (assessing understanding of the nature and the effect of voting), as well as ‘reasoning’ themes, in the modified questionnaire. The impact of this modification on the participants understanding of the phrasing of the questions or impact on overall scores was not established. This limits the usefulness of the results and any comparison with international studies that used an unmodified CAT-V, and any subsequent conclusions regarding voting competency. A larger validity and reliability study is needed.

Language served as a limiting factor as only those who were English-speaking were selected to participate.

## Conclusion

The findings from this study suggest that current legislative restrictions on voting registration for MHCUs may not be justified. Limitations such as the inclusion of both state patients and involuntary MHCUs, lack of use of cognitive screening tools and validation of the MCAT-V make it difficult for definite conclusions to be drawn. However, this study is the first to investigate voting knowledge and related decisions amongst hospitalised MHCUs in South Africa. Based on the Doe voting capacity standard, South African MHCUs do not have significantly different patterns of voting knowledge and choice demonstration when compared with a control group, but differences between the groups were noted on reasoning scores. The differences in total MCAT-V scores between the groups were further found to be associated with education level and not membership of the MHCU group. It is therefore recommended that because of the educational bias, this MCAT-V questionnaire in its current format should not be used as a screening tool in the South African setting. There is a need for further research, addressing the above limitations.

## Appendix

BOX 1-A1 Modified competence assessment tool for voting questionnaire. Imagine that there are two political parties participating in an election in South Africa:

1. What will the people of South Africa do in the election to pick a new ruling party? **(Assessing the understanding of the nature of voting)**
  **Score 2: correct answer    Score 1: partially correct answer    Score 0: incorrect answer**
2. When the election is over, how will it be decided who the new ruling party is? **(Assessing the understanding of the effect voting)**
  **Score 2: correct answer    Score 1: partially correct answer    Score 0: incorrect answer**
3. Political party A wants to put more money into SASSA grants. Political party B want to put more money into housing allowances.
  Which party would you vote for? **(Assessing the ability to choose)**
  **Score 2: clearly indicates choice    Score 1: choice is ambiguous    Score 0: unable to make a choice**
4. What is your reason for the choice in the question above? **(Assessing reasoning)**
  **Score 2: Able to identify one comparative attribute    Score 1: Ambiguous    Score 0: Unable to mention a comparative attribute**
5. If your choice of political party as mentioned above were to be elected, how would it affect your life? **(Assessing understanding of consequences)**.
  **Score 2: Able to identify a consequence    Score 1: Gives a vague consequence to his/herlife    Score 0: Unable to give a consequence**
6. Would you want to vote in the next South African General Elections? And why?
  **Score 2: Able to give a good reason for their answer    Score 1: Gives an ambiguous reason for their answer    Score 0: unable to adequately explain their answer**

*Source:* Permission was obtained for modification from the original designers of the CAT-V questionnaire. Appelbaum PS, Bonnie RJ, Karlawish JH. The capacity to vote of persons with Alzheimer’s disease. Am J Psychiatry. 2005;162(11):2094–2100. https://doi.org/10.1176/appi.ajp.162.11.2094
